# The Moyamoya Health Behavior Scale for Adolescent Patients: Measurement Tool Development and Psychometric Evaluation

**DOI:** 10.3390/ijerph18084064

**Published:** 2021-04-12

**Authors:** Won-oak Oh, Insun Yeom, Sung-Hyun Lim, Dong-Seok Kim, Kyu-won Shim

**Affiliations:** 1 College of Nursing, Korea University, 145 Anam-ro, Seongbuk-gu, Seoul 02481, Korea; wooh@korea.ac.kr (W.-o.O.); l_sh1676@naver.com (S.-H.L.); 2 Department of Pediatric Neurosurgery, Severance Children’s Hospital, Yonsei University Health System, 50-1 Yeonse-ro, Seodaemun-gu, Seoul 03722, Korea; dskim33@yuhs.ac (D.-S.K.); shimkyuwon@yuhs.ac (K.-w.S.)

**Keywords:** moyamoya disease, adolescent, health behavior, psychometrics, safety evaluation

## Abstract

Clinical practitioners treating moyamoya disease recognize the need for a systematic approach to better manage the disease in adolescent patients with the disease. *Methods*: This study aimed to develop and evaluate the validity and reliability of a disease scale which measures the health-related behaviors of adolescents with moyamoya disease. *Results*: The final 12-item Moyamoya-HB Scale for adolescents was categorized by three sub-domains: implementation of treatment for moyamoya disease (four items); health promoting behavior for moyamoya disease (four items); and health coping behavior for moyamoya disease (four items). Overall, these factors explained 68.97% of the total variance. The results of the confirmative factor analysis supported the construct, convergent and discriminant validity of the three sub-domains. The Moyamoya-HB Scale for adolescents also demonstrated a concurrent validity with the Korean Adolescents’ Health Behaviors Tool (r = 0.59, *p* < 0.001). Reliability analysis showed an acceptable-to-high Cronbach’s alpha of 0.865 in total, and the subscales ranged from 0.800 to 0.841. *Conclusions*: Initial findings support the Moyamoya-HB Scale as a reliable and valid measure of health behaviors in adolescents with moyamoya disease.

## 1. Introduction

Moyamoya disease is a rare chronic progressive cerebrovascular obstructive disease that forms an abnormal microcirculatory loop in the base of the brain due to stenosis or occlusion of one or both of the major branches of the Willis Circle [1,2,3].

The precise cause and mechanism of the disease have not yet been determined, but several epidemiological characteristics have been investigated. Moyamoya disease is more common in Northeast Asia, including Korea, Japan, China and Taiwan, than in Europe or North America. In Washington State, USA, for example, the prevalence rate is 0.086 per 100,000 [4], while the prevalence rate is 3.16 in Japan and 18.1 in Korea. In particular, the prevalence of moyamoya disease in Korea increased nearly threefold from 6.3 in 2005 to 18.1 in 2015. Although there are some differences among countries depending on gender, there is a higher incidence rate for females in Japan (1:1.18 male-to-female ratio) and Korea (1:1.43) [1]. The age-specific prevalence is 21.6% for 10–19 year-olds and 30% for 50–59 year-olds [2].

Many surgical treatment methods for the treatment of moyamoya disease have been developed [2,5]. These surgical treatment methods can be classified into two types: direct or indirect anastomosis. By performing such angioplasty, collateral blood circulation through the cerebral cortex can be increased, thereby reducing the frequency of ischemic attacks [6,7,8,9,10,11]. The disease has no cure, however [8,9,10].

The main clinical manifestations of moyamoya disease are life threatening neurological symptoms of transient ischemic attack (TIA), cerebral infarction and cerebral hemorrhage, which cause symptoms such as dyspepsia, speech disorders and abnormal sensations. Blood flow disorders in the brain lead to temporary or permanent brain dysfunctions such as seizures and higher brain dysfunction [11,12,13,14]. In particular, the most common triggering factors for cerebral ischemic attacks are related to hyperventilation, such as singing, blowing into a musical instrument, severe crying, excessive exercise and eating hot or spicy foods [15]. Also, adolescents with moyamoya disease cannot enjoy the same physical activities or lifestyle as their friends at the same age, depending on the characteristics of the disease [2]. This may be a result of accumulation of physical activity limitations related to the manifestation of symptoms, which may also result in experiencing personal stress, thus causing additional symptoms. In addition, cerebral ischemic attacks can occur in stressful situations. In this case, the symptoms immediately improve within a few seconds to a few minutes, but the longer the duration of the cerebral ischemia the more likely it will impact daily life, so stress management is required [6,15,16]. Therefore, health behaviors in the everyday life of adolescents with high stress are encouraged. 

The adolescent period is a transitional period in which development and normal physical, psychological and social changes are experienced in transition, and health behaviors formed during this period have a significant effect on adulthood health status [17]. It is very important to develop a strategy for promoting health. Adolescence is also the most physically healthy period in the life cycle and the beginning of the greatest number of health risk behaviors [18,19,20,21]. In particular, when adolescents are diagnosed with chronic diseases such as adolescent moyamoya disease, physical and mental stress increase due to the diagnosis [22]. In the end, this stress can lead to a vicious cycle in which brain ischemic attacks, which are the main symptom of moyamoya disease, occur more frequently.

To promote normal growth and development, adolescents with moyamoya disease need specific intervention strategies that can mitigate the effects of their disease, namely, moyamoya disease-specific management and healthy behaviors. In order to achieve this, an accurate understanding of the health-related behavior of adolescents with moyamoya disease is necessary. Based on these results, it can be essential to set goals and plan programs for promoting healthy activities for adolescents with moyamoya disease [9]. The results can also be linked to program effectiveness assessment and ongoing monitoring systems. Accurate and well-documented data can contribute to the efficient use of limited resources and ultimately promote healthy behaviors that are desirable for adolescents with moyamoya disease [5,23].

However, research on adolescent moyamoya disease is very limited, and there are no measurement tools to identify health-related behavior for adolescents with moyamoya disease. For example, we provide education to patients and their parents by conducting dis-ease-related courses and handing out brochures [1,5].

However, in contrast to the recognition of the importance of daily life management in mitigating the ill effects of moyamoya disease in several studies, there is a lack of a clear protocol for disease-related healthy behaviors, and there is a lack of a clear scale and lack of motivation. Nevertheless, clinical practitioners treating moyamoya disease recognize the need for a systematic approach to better manage the disease in patients with moyamoya disease.

In particular, adolescents with moyamoya disease experience many psychological and physical changes according to their developmental transitional characteristics, and it is necessary to manage clinical symptoms well through the implementation of disease-related healthy behaviors.

Therefore, before designing systematic interventions (education and programs) for the maintenance of the health of adolescents with moyamoya disease, it is necessary to clearly define and measure the health behavior of moyamoya disease so that patients can perform essential and practical actions. In other words, understanding of the characteristics of moyamoya disease and management of major risk factors such as respiration, disease-related psychology and emotional control are needed. It is necessary to develop a tool to measure specific health hazards related to moyamoya disease that can specifically measure health behaviors and behaviors that should occur when symptoms occur. 

The purpose of this study was to develop and evaluate the validity and reliability of a moyamoya disease scale which measures the health-related behaviors of adolescents with moyamoya disease.

## 2. Materials and Methods

### 2.1. Research Design

**Figure 1:**
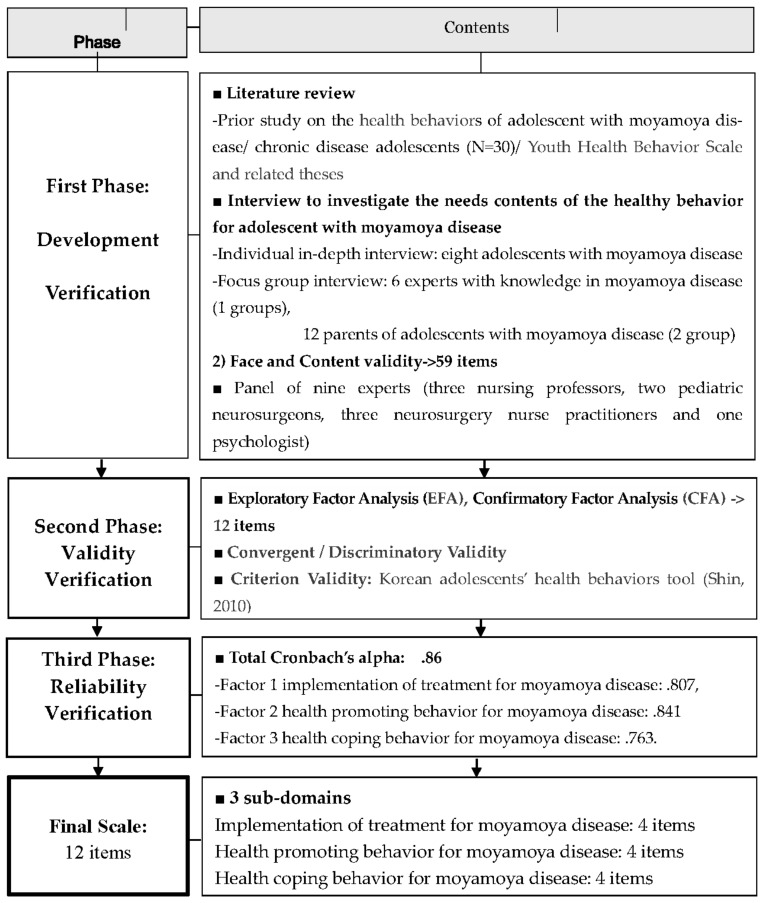
Process of program development and psychometric testing.

Figure 1 summarizes the research design used in this study.

### 2.2. Development Verification of the Moyamoya-HB Scale 

The three major phases of developing the Moyamoya-HB Scale (item generation, content validity testing and item analysis) were performed as reported by DeVellis [24].

#### 2.2.1. Item Generation Stage: Identification of Preliminary Questions

##### Review of Literature and Consideration of Existing Tools

We reviewed domestic and foreign literature and youth health-related scales published before 2017 in order to construct sub-domains of health-related behavior of adolescents with moyamoya disease. The key phrases “adolescent health behavior”, “moyamoya disease health behaviors” and “moyamoya disease adolescent health promotion” were used for searches using PubMed and the Korea Education and Research Information Service (KERIS). As a result, 30 articles were screened. In addition, we reviewed six aspects of the Shin [25] Youth Health Behavior Scale and related theses. As a result, we classified adolescent health activity domains into the responsibility for one’s health domain, physical activity domain, nutrition domain, psychological domain, interpersonal domain and stress management domain. However, these areas were found to have limitations in measuring health-related behaviors associated with moyamoya disease after we conducted in-depth interviews.

##### In-Depth Interviews for Initial Questions

In-depth interviews were conducted with eight adolescents with moyamoya disease and 12 parents. Group interviews were conducted by six experts (two pediatric neurosurgeons, one psychologist and three nurses). Participants for this study were recruited from the ward and outpatient clinic of the Pediatric Neurosurgery Department at Yonsei University Hospital in Seoul, South Korea. The inclusion criteria for moyamoya disease with adolescents were: (a) be 14 to 19 years old; and (b) have been diagnosed with moyamoya disease for more than one month. The exclusion criteria for patients were a history of mental illness or difficulty in participating in the interview (e.g., hearing impairment, visual impairment, etc.). Interviews were conducted with parents of participating adolescents with moyamoya disease, and six of the patients who participated in the study were interviewed. One-to-one in-depth interviews using semi-structured questions were conducted from July to September 2017, and group interviews were conducted from August to September 2017. Each was performed for about 40 to 70 min. The interviewees were asked: (1) “What is the most important question to ask related to health behaviors of adolescents with moyamoya disease?”; (2) “What behaviors of adolescents with moyamoya disease maintain and promote their health?”; (3) “What behaviors are risky behaviors?”; and (4) “What behaviors prevent these adolescents with moyamoya disease from maintaining and promoting health?” The qualitative data collected through the interviews were analyzed at the same time as data were collected, and the conventional qualitative content analysis method was used [26]. We used reliability, applicability, consistency and neutrality according to the qualitative research criteria of Lincoln and Guba [27] for the trustworthiness of the interview data.

#### 2.2.2. Content Validity Testing: Face and Content Validity

The preliminary questions were judged by a panel of nine experts (three nursing professors, two pediatric neurosurgeons, three neurosurgery nurse practitioners and one psychologist who has more than 10 years of experience in psychological counseling for patients with moyamoya disease). The experts scored each item in terms of its significance and logical consistency to the tool.

The Content Validity Index (CVI) is the most widely reported approach for content validity in instrument development and can be computed using the Item CVI [28]. The experts scored each item ranging from 1 to 4 with a four-degree range of “1 = not relevant, 2 = somewhat relevant, 3 = quite relevant, and 4 = very relevant”, respectively, and in terms of the logical consistency of all items to the overall scale. Values range from 0 to 1 where I-CVI > 0.79 showed the item is relevant, between 0.70 and 0.79 the item needs revisions, and if the value is below 0.70 the item is eliminated [28].

#### 2.2.3. Item Analysis

Item analysis is considered the second-most important part of the scale development process after item generation [29]. The item analysis was conducted with (a) a corrected item-to-total correlation coefficient and (b) an inter-item correlation matrix, and (c) the item-to-total correlation coefficient was selected from 0.30 to 0.80 [30] to assess the contribution of items and avoid collinearity. The final 25 selected items were put into EFA and CFA analysis for validity and reliability verification.

### 2.3. Tests of Validity 

After analyzing the questionnaire, exploratory factor analysis (EFA), confirmatory factor analysis (CFA), item convergence and a validity test were conducted to validate the construct of the Moyamoya-HB tool. The criteria-related validity was confirmed using concurrent validity analysis. 

#### 2.3.1. EFA and CFA

Exploratory factor analysis (EFA) is a method of generating a model or structure by exploring features inherent in the data without special assumptions about the number or structure of the tools [29]. The items of the preliminary tools developed in this study were developed without any assumptions about structure. Factor analysis of the items is a more detailed study of whether the correlations between the items justify believing that the items measure the same trait. If the items turn out not to be unidimensional, the scale might have to be split into subscales, or items might have to be removed (Kaiser-Meyer-Olkin, KMO, value > 0.6 and Bartlett test results showing significant sphericity) [31]. Therefore, by exploratory factor analysis, the number of factors and structure of the health behavior measurement scale of adolescents with moyamoya disease were confirmed. After EFA, CFA was conducted to verify whether the collected data and the stability of the factor structure. The goodness of fit was evaluated using Chi-square/df, root-mean-square error of approximation (RMSEA), comparative fit index (CFI) and IFI (Incremental Fit Index).

#### 2.3.2. Convergent/Discriminatory Validity

A multi-trait/multi-item matrix analysis was conducted to examine the convergent and discriminant validity of the items. The questionnaire verified the convergent validity of the items by checking whether the items overlapped with the sub-domains in which each item was included and the correlation coefficient with the total score of the calculated sub-domain was at least 0.40. The validity of each item was shown to be highly correlated with the sub-domain in which each item of the developed tool subtracts itself from its sub-domain and had a low correlation to distinguish it from the domain other than the sub-domain. The correlation coefficient between the sub-region and the other region is greater than twice the standard error of correlation coefficient [32].

#### 2.3.3. Criterion Validity

The researchers used the concurrent validity test method to verify the validity of the developed tools. The generalized Korean Adolescents’ Health Behaviors Tool was used at the time of development based on the opinions of moyamoya disease experts who thought it would be the most appropriate tool, as it has been verified for its usefulness in other studies as a measuring tool for adolescents’ health behavior (Chronbach’s Alpha > 0.85). In order to test the concurrent validity of this tool, the correlation with the Korean Adolescents’ Health Behaviors Tool [25] was a total of 72 items, and 14 factors of health behavior. Each questionnaire used a 4-point Likert scale. 

However, in order to measure the validity of the tools of this study, items such as sexual intercourse, hygiene, food intake and weight, which were not directly related to health behavior associated with moyamoya disease, were excluded. Cronbach’s value of all instruments at the time of development was 0.82 and the test—retest correlation coefficient was 0.85. The alpha value in this study was 0.81.

### 2.4. Test of Reliability

Reliability testing for the scale uses the Cronbach alpha score to confirm the internal consistency. A Cronbach’s a value of between 0.70 and 0.80 indicates that internal consistency reliability is good, 0.80 and 0.90 is very high, but 0.90 and above based on the DeVellis [24] criterion suggests that the number of questions should be reduced. Cronbach’s α was calculated for the entire Moyamoya-Health Behavior scale (Moyamoya-HB scale) and for each extracted factor. 

To confirm the stability, adolescents with moyamoya disease were asked to participate in a test–retest survey and to provide their contact information if they consented. Then, Pearson’s test–retest correlation coefficient was verified. 

The evaluation of the Moyamoya-HB Scale was conducted by Yonsei University Hospital in Seoul, Korea. The study subjects were made to understand the purpose of the study and agreed to participate. The expected number of subjects using G * Power [33], a sample size program according to Cohen’s sampling formula, was set at significance level 0.05, power of 0.80 and effect size of 0.30 for 128 persons. The initial data were collected from 130 persons, but inappropriate data were removed. A total of 120 subjects were included in the analysis of the final data of this study.

### 2.5. Ethical Considerations

This study was conducted after ethics approval by the Yonsei University Health System, Severance Hospital., Institutional Review Board. Interview participants, survey participants and caregivers were asked to disseminate explanations of the purpose and method of the study to the study participants’ and received their consent. The consent form stipulated that the personal information acquired through this study would be used only for research purposes and that the subject freely participated in the study and could withdraw from the study at any time.

### 2.6. Data Analysis

The analysis was conducted using IBM SPSS/WIN 22.0 program (SPSS, Chicago, IL, USA) and AMOS version 20 (SPSS Inc., an IBM Company, Chicago, IL, USA). Exploratory factor analysis (EFA) was performed by using principle component analysis with varimax rotation. In conducting confirmatory factor analysis (CFA), the model fit was verified on the basis of the chi-square test, normal fit index (NFI), comparative fit index (CFI), root-mean-square error of approximation (RMSEA), the goodness of fit index (GFI) and the standardized root mean square residual (SRMR). Reliability coefficients were calculated to verify reliability, stability, homogeneity and internal consistency. In addition, the Pearson correlation test was used to examine the correlation between scores from the Moyamoya-HB Scale and the Korean Adolescents’ Health Behaviors Tool [25] to test for concurrent validity among criterion-related validity.

## 3. Results

### 3.1. Item Generation

Based on the conceptual framework and constituent factors, the initial criteria of the tool were constructed on the basis of previous research, existing measurement tools and interview data. A total of 102 statements were made and a total of 65 initial questions were developed by integrating similar statements. The initial questions consist of nine domains: lifestyle, physical activity, health coping, future design, safety, nutritional habits, social support, mental health and moyamoya treatment. The physical activity domain consisted of activities related to the disease and regular exercise. The health coping domain includes emergency coping related to the disease and the control of the risk factors, and future design consists of activities for the positive stage of development in adolescence. The safety and nutrition habit domains are composed of specific general health safety guidelines. The social support and mental health domains consisted of healthy activities to utilize their resources and positive understanding of the meaning of life and stress management. The range of treatment included regular check-ups, medication and daily life guidance implementation, which was deemed necessary to confirm the implementation of treatment instruction through the review of the literature [34,35]. Statements consisted of answers from the 5-point Likert scale (e.g., one point for “not at all” to five points for “always”).

In order to verify the validity of the results, nine people, including two pediatric neurosurgeons, three pediatric nursing professors, two nursing specialists in pediatric neurosurgery, one psychologist and one resident, formed a team. The items were revised and supplemented according to the opinions of the experts, and the deletion and addition of items that were not related to the health behavior of adolescents with moyamoya disease were done. The CVI results of the total 65 items and the item level content validity index of the 59 items were 0.90~1.00, which was above the reference value of 0.78 [36]. As a result, a total of 59 preliminary questions were identified for the evaluation phase of the Moyamoya-HB tool.

### 3.2. Content and Face Validity

Nine experts participated in phase 2 to test the face and content validity (response rate = 100%). This team included three nursing professors (each with more than 15 years of teaching experience), two pediatric neurosurgeons (each with more than 20 years of clinical experience with patients with moyamoya disease), three neurosurgery nurse practitioners (each with more than 10 years of clinical nursing experience) and one psychologist. 

The first content validity round was assessed by CVR, I-CVIs indicating that only 59 items on the 65 proposed by the initial items achieved adequate content validity CVRs higher than 0.70 [28].

Consequently, the results of the comments for face validity confirmed the over plus of those items with low CVR and I-CVI (Item-Content Validity Index). As suggested by the experts, several items were removed for improved comprehensibility, and some items were integrated, which resulted in 59 items.

### 3.3. General Characteristics of Participants

The mean age was 15.00 (standard deviation = 1.60), and the mean follow-up was conducted 35.64 (standard deviation = 30.94) months later. The general characteristics of the additional diseases were as follows (Table 1).

### 3.4. Item Analysis 

In order to confirm the cluster possibility and contribution of the items, we conducted a questionnaire consisting of the 59 items that were tested through content validity. As a result, we eliminated 34 items such as “I do not depend on sleeping pills”, “I do not eat”, “I do not drink”, “I eat breakfast”, “I am not afraid of a new experience”, “I am not afraid of new experience” and “I maintain a proper weight” that were more than 75% in correlation with a value less than 0.30 [30]. In addition, these 34 items also correlated to 0.35~0.40 in the item-to-total correlation coefficient analysis [31], and the 25 remaining items were properly confirmed as 0.51~0.80, and 34 items were removed from the item analysis.

### 3.5. Validity Verification

#### 3.5.1. Exploratory Factor Analysis (EFA)

In order to test the adequacy of the EFA, the KMO and Bartlett’s KMO (Kaiser-Meyer-Olkin) values were found to be higher than 0.509 for the 25 items identified in the item analysis of this study. Also, Bartlett’s chi-square test of sphericity (χ^2^ = 3586.07, *p* < 0.001), and the validity of the analysis was established in EFA [31]. Factor analysis was performed using principal component analysis and the orthorectic rotation of Varimax. The eigenvalues of each factor were scaled by one or more and scree plotted according to Kaiser’s rule application. The commonality of each item was 0.20 or more. The common variance is the part where each item is explained by a common factor. If the value is less than 0.20, the variance explained by the common factor is considered to be small, which means that the factor coefficient is highly correlated to only one factor [37]. In addition, there is no absolute criterion to judge the significance of the factor loadings of the items [38], but it is suggested that 0.40 is a conservative level of significance [39]. In this study, we selected the items with an absolute value of factor load of 0.60 or more.

As a result of the first factor analysis, three factors were extracted. Thirteen items with an absolute value of factor load less than 0.60 were deleted and one item with factor complexity was included in the other items to measure similar concepts. The first 12 items were selected.

Three factors were extracted by the second factor analysis for these 12 questions. Factor analysis of the tool by Varimax rotation showed that three factors were extracted in the same way as the factor with an eigenvalue of one or more. The scree plot also shows a nearly horizontal change after the third eigenvalue decline. Table 2 shows that the explanatory variables of the three factors were 22.3% for the first factor, 21.7% for the second factor, and 19.8% for the third factor (Table 2).

Factor 1 is “Treatment Instructions for Moyamoya Disease”, which consists of items corresponding to the implementation of healthy behavior in the process of treating moyamoya disease. Factor 2 is named as “Moyamoya Health Promotion Acts”, consisting of items corresponding to healthy life and habits associated with moyamoya disease in order to maintain and promote health. The third factor is related to the illness coping behaviors of adolescents with moyamoya disease, mainly coping with risk factors and coping behaviors related to clinical symptoms. The Factor 3 is health care items related to illness characteristics called “Health coping behavior for moyamoya disease”.

#### 3.5.2. Confirmatory Factor Analysis (CFA)

**Figure 2:**
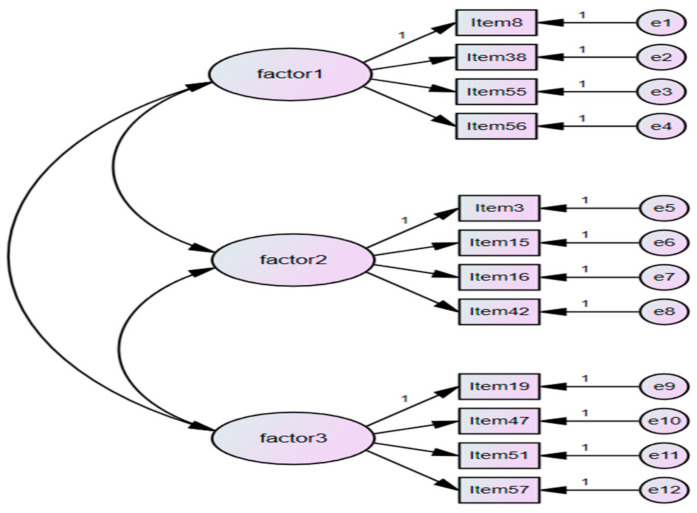
Confirmatory factor analysis.

Confirmatory Factor Analysis (CFA) was conducted to elicit more precise results on the validity of the Moyamoya-HB Scale with 12 items. Indices of χ^2^, GFI, CFI, IFI, SRMR, and RMSEA were used to determine the adequacy of fit of the model. The χ^2^ = 763.95 was not significant (*p* < 0.001). However, because the χ^2^ statistic is highly sensitive to sample size and may overstate the lack of a fit of a model [40], the fit of the model should be interpreted on the criteria of other indices, such as normed χ^2^ < 3, GFI > 0.90, CFI > 0.90, NFI > 0.90, SRMR < 0.05, and RMSEA < 0.08. Therefore, the HSS-Kids model showed a good fit because it met, or approximated, all additional indices criteria (Figure 2).

#### 3.5.3. Concurrent Validity

In order to test the concurrent validity of the Moyamoya-HB scale, we did a correlation with the Korean Adolescents’ Health Behaviors Tool. We found there was a statistical correlation (r = 0.59, *p* < 0.001) between health behavior of adolescents using both tools.

#### 3.5.4. Convergent Validity and Discriminant Validity

The results of this study were as follows: (1) The standardized factor loadings for each item for convergent validity of health-related behavior items of adolescents with moyamoya disease should be at least 0.50 and over 0.70 should be preferred [39]. All the items in the study were found to meet the minimum criteria except for one item, and all the remaining seven items were found to be more than 0.70 (Table 3).

As an alternative method for discriminative validity, we calculated the variance extraction index based on the standardized fit of the latent variables and the error of the measured variables. Conceptual reliability refers to the degree of coherence between measured variables, which indicates the shared variance among the measured variables of a constituent concept. The results of confirmatory factor analysis were calculated from the derived factor loadings and the error variance (Table 3).

When the variance extraction index of the corresponding latent variable is greater than the square of the correlation with all other factors [41], the correlation coefficient between Factor 1 and Factor 3 was 0.609 ((0.609)^2^ = 0.371) when ‘Factor 1<->Factor 3’ was applied, and the average variance extracted (AVE) of Factor 1 was 0.510 and AVE of Factor 3 was 0.543. Both AVE values were greater than the square of the correlation coefficient.In order to determine the discriminant validity of the Moyamoya-HB scale, the correlation coefficient and correlation standard error $\left(\sqrt{1-r^{2}}/n-2\right)$ between factors were calculated. A discriminant validity is established when the confidence interval of the correlation coefficient $\left(r-2\times S\cdot E\sim r+2\times S\cdot E\right)$ is less than one [40]. The correlation coefficient of Factor 2 and Factor 3, which showed the highest correlation coefficient, was calculated as 0.863~0.905, and it was judged to be valid because it was less than one. The confidence interval of the correlation coefficient between the other factors was less than one as well. As a result, the discriminant validity of Moyamoya-HB Scale was confirmed.

To confirm the reliability of Moyamoya-HB scale, the AVE (≥0.5) and construct reliability (≥0.7) were calculated of CFA (37) and Cronbach’s alpha coefficients (Table 3). As the AVE ranged from 0.453 to 0.798, and construct reliability ranged from 0.763 to 0.824, the reliability of all factors in the Moyamoya-HB Scale was confirmed.

### 3.6. Reliability Verification

For the 12 items of the Moyamoya-HB scale, Cronbach’s alpha values for the overall scale, and the subscales were high. The total Cronbach’s alpha was 0.86; for Factor 1 (implementation of treatment for moyamoya disease) it was 0.807, for Factor 2 (health promoting behavior for moyamoya disease) it was 0.841, and for Factor 3 it was (health coping behavior for moyamoya disease) 0.763.

## 4. Discussion

As a result of the study, the Moyamoya HB-scale for measuring health behaviors of adolescents with moyamoya disease developed 12 items in threesub-domains: implementation of treatment for moyamoya disease, health promoting behavior for moyamoya disease and health coping behavior for moyamoya disease, all on a 5-point Likert Scale. Factor 1, which is “implementation of the treatment of moyamoya disease” consisting of four items, has the second most powerful explanatory power (22.78%).

This is a core activity that should be performed during the treatment of moyamoya disease. In other words, it corresponds to the behavior associated with essential clinical prescriptions in the treatment of moyamoya disease [42,43,44]. Water intake is important for patients with moyamoya disease because dehydration can cause neurologic (clinical) symptoms [1,42,45]. Adolescents with moyamoya disease also spend a lot of time at school, and thus treatment instructions should be learned for emergency situations that may occur at school. Also, regular check-ups should be done and basic rules for taking medication should be followed in the course of treating moyamoya disease. This can be seen in the same context as Kim [46]’s conclusion that in order to prevent cancer in high school students, the implementation of health behaviors is more important than the knowledge of health behaviors.

The second factor, at 24.54%, was the most important factor in the measurement of health behaviors among adolescents with moyamoya disease. This factor, “health promoting behavior for moyamoya disease”, includes healthy behavior and habits which maintain and promote the health of adolescents with moyamoya disease. In other words, we were able to measure posture in order to keep the cerebral blood circulation healthy, essential items to be brought in when going out, useful information collection for disease management strategy and support for specific health promotion through information networks. This is seen as a similar concept to self-health promotion behaviors such as health education, social care, and disease prevention in other group health behavior measurement tools [46]. In addition, in Byun and Lee’s [47] research on adolescents’ health behaviors, behaviors contrary to health promotion are seen in the same context as those viewed as health risk behaviors in adolescents.

The third factor was “explanatory power” (21.48%), and the term “health coping behavior for moyamoya disease” relates to adolescents with moyamoya disease taking appropriate action related to the disease. That is, behaviors related to management of stress, hyperventilation and mental health, because stress can cause clinical symptoms, and ineffective resolution of stress can also affect individual health behaviors, including negative emotions such as depression and anxiety. Also, negative emotions can cause stressful situations. This result is the same as seen by as Oh and Bae [46], who reported that adolescents’ active stress coping influenced positive emotions, and did not induce risk behaviors such as Internet addiction.

The purpose of this study is to develop a tool to measure health behaviors in consideration of the characteristics of illnesses of adolescents with moyamoya disease. It is also important to verify the validity and reliability of the Moyamoya-HB Scale from other studies to provide strong evidence that can be used as a useful tool in various research fields. In the development of tools, it is necessary to present a rational basis for tool use by presenting diverse and logical analysis for validating the tool [48].

The reliability of the developed tool was tested by measuring the degree of internal consistency among items considering the characteristics of adolescents with moyamoya disease. It was confirmed to be reliable [48] with a Cronbach’s α value of 0.86, which shows high internal reliability, proving this to be a highly reliable tool.

In addition to verifying the validity and reliability of the Moyamoya-HB scale, this study has the following significance. The Moyamoya HB-scale, consisting of sub-domains of cognition, practice and emotion, maintains a relatively high explanatory variance of 68.97%. In the first factor analysis, the 12 items were reduced from 25 items, but maintaining a relatively high explanatory variable means that the construct validity should not be threatened due to the reduction of the items in the tool development [49]. Second, there is significance as the first development of health behaviors of adolescents with moyamoya disease, a rare incurable disease. It is also important as a basic study for programs for adolescents with moyamoya disease that should be studied in the future.

It is expected that the development and application of this Moyamoya-HB Scale will provide basic data on the development of various intervention strategies for the health promotion of moyamoya disease adolescents in nursing and medical care environments. In addition to the significance of the above study, in order to overcome the restriction of the recruitment of subjects through a hospital and the disease consultation, we tried to satisfy the construct validity through various methods.

## 5. Limitations

This study has some limitations. First, the subjects self-reported their subjective responses to each item in the questionnaire during the validity and reliability phases. Given that there may be differences in the consistency of responses, there are limitations in this method of investigation. This suggests follow-up studies of additional confirmatory factor analysis should be conducted. Second, it is suggested to investigate the applicability of the Moyamoya-HB Scale with other diseases. Third, the Moyamoya Health Behavior Scale for adolescent patients developed in this study is a tool developed and verified for Korean adolescents with moyamoya disease. Therefore, it is suggested that a study on the reliability and validity of each country’s scale will be required for this tool in the future.

## 6. Conclusions

The Moyamoya-HB Scale was developed to evaluate and verified as an acceptable assessment tool to measure health behavior of adolescents with moyamoya disease through a variety of psychometric evaluations. It is a self-report form tool composed of three sub-domains: implementation of treatment for moyamoya disease, health promoting behavior for moyamoya disease and health coping behavior for moyamoya disease, using a 5-point Likert scale. The purpose of this study was to identify the essential elements of health behaviors of adolescents with moyamoya disease when considering the health characteristics that are important for health maintenance and life management. It is meaningful for developed and evaluated tools to be available. We hope that this study will be useful as a theoretical basis and evaluation index for the development and application of various intervention programs for health promotion of adolescents with moyamoya disease.

## Figures and Tables

**Table 1:** Characteristics of participants (*n* = 120).

Variable	Category	Number of Patients (%)	Mean (±SD)	*p*-Value *
Sex	Male	71 (59.17)		0.02 *
	Female	49 (40.83)		
Age			15.00 (±1.60)	
Follow-up (month)			35.64 (±30.94)	
Suzuki stage			3.18 (±0.54)	
Diagnostic path	Clinical symptoms	114 (95.00)		0.19
	Medical checkup	6 (5.00)		
	During examination for other diseases	0 (0.00)		
Disease severity	Very serious	6 (5.00)		0.03 *
	Serious	34 (28.33)		
	Average	56 (46.67)		
	Not so serious	24 (20.00)		
	Not at all serious	0 (0.00)		
Influence on normal life	None	10 (8.33)		0.04 *
	Slight	74 (61.67)		
	Some	13 (10.83)		
	Moderate	23 (19.17)		
	Severe	0 (0.00)		
Frequency of symptom (last month)	Never	6 (5.00)		0.03 *
	Rarely	44 (36.67)		
	Sometimes	32 (26.67)		
	Very Often	32 (26.67)		
	Constant	6 (5.00)		
Cerebral hemorrhage/cerebral infarction (history)	Infarction	27 (22.50)		0.04 *
	Hemorrhage	0 (0.00)		
	Both	0 (0.00)		
	Neither	93 (77.50)		
Seizure	Yes	113 (94.17)		<0.05 *
	No	7 (5.83)		
Experienced TIA	Yes	119 (99.17)		<0.05 *
	No	1 (0.83)		
TIA more than once a month	Yes	91 (75.83)		0.03 *
	No	29 (24.17)		
Surgery (EDAS)	No	2 (1.67)		0.04 *
	Both	86 (71.67)		
	One	32 (26.67)		

SD: Standard Deviation. TIA: Transient Ischemic Attack. EDAS: Encephalo-duro- arterio- synangiosis. * *p*-value < 0.05 is significant.

**Table 2:** Factor Loading of Questionnaires.

Factors	Item Card No.	Communalities	Factor Loading		
			Factor 1	Factor 2	Factor 3
Factor 1 (Implementation of treatment for moyamoya disease)	health08	0.660	0.656	0.405	−0.256
	health38	0.809	0.838	0.139	0.296
	health55	0.698	0.803	0.122	0.195
	health56	0.687	0.774	−0.062	0.290
Factor 2 (Health promoting behavior for moyamoya disease)	health03	0.613	0.308	0.673	0.257
	health15	0.799	0.058	0.861	0.232
	health16	0.781	−0.027	0.859	0.205
	health42	0.564	0.147	0.721	0.151
Factor 3 (Health coping behavior for moyamoya disease)	health19	0.725	0.308	0.361	0.707
	health47	0.561	0.205	0.081	0.716
	health51	0.713	−0.028	0.241	0.809
	health57	0.667	0.315	0.308	0.688
Explained variance		8.276	2.733	2.944	2.599
Explained (%)		68.974	22.778	24.535	21.661

**Table 3:** Convergent/Discriminant Validity and Reliability from CFA.

Theoretical Variables	Measurement Variables	Estimated λ	Standard Error	Standardized Estimate	C.R.	*p*	SMC	AVE	Construct Reliability	Chronbach’s Alpha	* SMC (** CC)
Factor 1 (Treatment Instructions for Moyamoya Disease)	Item08	1.000	-	0.494	-	-	0.244	0.510	0.798	0.807	Factor 1 & Factor 2 0.102 (0.320)
	Item 38	2.033	0.354	0.946	5.742 ***	<0.001	0.895				
	Item 55	1.417	0.273	0.750	5.199 ***	<0.001	0.563				
	Item 56	1.162	0.231	0.707	5.032 ***	<0.001	0.500				
Factor 2 (Moyamoya Health Promotion Acts)	Item 03	1.000	-	0.654	-	-	0.428	0.544	0.824	0.841	Factor 2 & Factor 3 0.371 (0.609)
	Item 15	2.172	0.275	0.905	7.893 ***	<0.001	0.820				
	Item 16	1.720	0.233	0.834	7.392 ***	<0.001	0.696				
	Item 42	1.426	0.228	0.651	6.266 ***	<0.001	0.424				
Factor 3 (Health coping behavior for moyamoya disease)	Item 19	1.000	-	0.863	-	-	0.745	0.543	0.763	0.800	Factor 1 & Factor 3 0.371 (0.609)
	Item 47	0.594	0.090	0.587	6.590 ***	<0.001	0.345				
	Item 51	1.134	0.159	0.628	7.144 ***	<0.001	0.394				
	Item 57	1.095	0.109	0.831	10.07 5 ***	<0.001	0.690				

* SMC = Squared Multiple Correlation. ** CC = Correlation coefficient. *** *p* < 0.001.

## Data Availability

The data that support the findings of this study are available from the corresponding author upon reasonable request.
